# Oxygen-enriched air reduces breathing gas consumption over air

**DOI:** 10.1016/j.crphys.2022.01.007

**Published:** 2022-01-30

**Authors:** J.D. Schipke, A. Deussen, F. Moeller, U. Hoffmann, T. Muth, A. Zenske, A. Koch

**Affiliations:** a Research Group Experimental Surgery, University Hospital Düsseldorf, Germany; b Institute of Physiology, Medical Faculty Carl Gustav Carus, TU Dresden, Germany; c German Sport University Cologne, Institute of Exercise Training and Sport Informatics, Department of Exercise Physiology, Cologne, Germany; d Institute of Occupational, Social and Environmental Medicine, Heinrich-Heine-University, Düsseldorf, Germany; e Klinikum Landshut, Operative Intensivmedizin und Schmerztherapie, Landshut, Germany; f German Naval Medical Institute, Kiel, Germany

**Keywords:** Exercise, Ventilation, Diving, Hyperoxia

## Abstract

Owing to the unfamiliar environment, recreational and professional diving is confronted with several challenges. Usage of self-contained under-water breathing apparatuses during the dive provides the indispensable breathing gas supply for the diver. Instead of air, oxygen-enriched breathing gases (EANx or nitrox) are used with increasing frequency. Unfortunately, their usage implies negative effects because the elevated oxygen partial pressure (pO_2_) increases oxidative stress. As a result, the increased formation of reactive oxygen species exerts negative effects on the central nervous system, lungs, vasculature and eyes. However, these disadvantages can be avoided if appropriate rules are followed, e.g. a pO_2_<1.4 bar. EANx breathing gases have, on the other hand, major advantages as they help reducing narcotic nitrogen effects and bubble formation.

Several land-based studies had proven a reduced ventilation of exercising subjects if EANx was used instead of air. As breathing gas is the most valuable under-water good, we wanted to translate the on-land results into under-water results. Appropriate studies now demonstrate a novel EANx property as under-water ventilation is also reduced with EANx. In this short communication, we present this additional advantage of EANx-breathing. This benefit seems to be of particular importance as it delays unforeseen running-out-of-gas and thus, contributes to further improving diving safety.

## Highlights

• Several ‘on-land’ studies had proven a reduced ventilation of exercising subjects in hyperoxia.
• As O_2_-enriched breathing gas (ANx) is increasingly used published on-land results were translated into under-water results.
• We demonstrate a novel EANx characteristic as under-water ventilation is also reduced with EANx compared with using air.
• This benefit is seemingly of importance as it delays unforeseen running-out-air. It thus contributes to diving safety.

## 1 Oxygen-enriched air

Possibly, Henry Fleuss made in 1874 what was the first oxygen-enriched air (=EANx or nitrox) dive using a rebreather (Lang, 2006). More than 100 years later, teaching nitrox use for recreational diving was begun in spite of heavy scepticism by the diving community. Still, the British Sub-Aqua Club started in 1995 nitrox training (Allen, 1996). More than 25 years later, nitrox has become the second most-used breathing gas besides air in recreational diving (Möller et al., 2021). Hence, quite a few effects of EANx on professional and recreational divers are well described.

In consequence to the increased oxygen partial pressure (pO_2_), oxidative stress exerts acute negative effects on the lungs (Doering et al., 2018) (Zenske et al., 2019) that are reversible (Tetzlaff and Thomas, 2017) (Castagna et al., 2019). In line, regular diving with nitrox at shallow depths over a 3-yr period did not impair pulmonary function (Fitzpatrick and Conkin, 2003). Conversely, oxidative stress could ultimately lead to non-reversible pulmonary pathological changes (van Ooij et al., 2013).

Oxidative stress has also been postulated to importantly contribute to central nervous system (CNS) oxygen toxicity (Kot et al., 2003). During dives a pO_2_ > 1.4 bar can ultimately lead to unconsciousness and convulsions (Harabin et al., 1995), while convulsions at a pO_2_ < 1.3 bar have not been reported (Arieli et al., 2002). Yet, factors like physical activity might modify the susceptibility towards CNS oxygen toxicity (Koch et al., 2013).

One other target constitutes the vascular system. Already a single air dive causes transient endothelial dysfunction (Culic et al., 2014). Expectedly, the flow mediated dilatation was significantly reduced after nitrox diving and after HBO-therapy, thus, arterial stiffness was increased (Marinovic et al., 2012) (Zenske et al., 2019) (Wunderlich et al., 2017).

Among other organs, the eyes can also be affected, and divers should be aware of the risk of lenticular oxygen toxicity when conducting intensive diving with a PO_2_ between 1.3 and 1.6 ATM. (Butler et al., 1999) (Brügger et al., 2020).

In contrast, the decreased partial nitrogen pressure (pN_2_) of nitrox exerts beneficial effects, particularly with respect to reducing N_2_ bubble formation after simulated (Souday et al., 2016) and real dives (Marinovic et al., 2012) (Brebeck et al., 2018), thereby reducing the risk of decompression sickness. In addition, nitrox could protect against decreased neuro-cognitive performance induced by inert gas narcosis (Germonpre et al., 2017) (Lafere et al., 2019) (Rocco et al., 2019), and so help to maintain alertness and memory (Brebeck et al., 2017). Thus, nitrox could be the breathing gas of choice in situations with the need for precise decisions or accurate assessments and actions.

In the following, one novel beneficial effect of nitrox breathing will be presented.

## 2 Breathing gas consumption

Minute ventilation (V̇E) is a crucial factor for both the duration and safety of a dive, and sudden running-out-of-gas is one among other factors associated with diving fatalities (Vann and Lang, 2011). Such a dangerous situation might develop due to an unexpectedly high ventilation during fin-swimming at depth and might result in an emergency ascent and severe diving accident. One other factor is mentioned that exposes the diver to a risk and is related to ventilation, as well. Some of the divers perform skip breathing to conserve their breathing gas and try to dive as long as possible with their limited gas supply. However, due to prolonged dive times, the risk of decompression increases. In addition, CO_2_ is retained, and the increased CO_2_ levels might lead to CNS oxygen toxicity (Arieli et al., 2014).

To answer the question, whether hyperoxia has any effect on V̇_E_, results from land-based studies are presented, first.

In an older, extremely worth reading review article on V̇_E_, the author concludes:

‘*… the data appear to indicate that in animal experiments respiratory minute volume is diminished by breathing O*_*2*_*; the same may be expected to be true for man but the data … are not unequivocal and for this one may perhaps question to what extent, if any, psychological factors may be responsible’* (Bean, 1945).

In a more recent meta-analysis on the same topic, 40 manuscripts on the effects of acute hyperoxia on healthy subjects exercising on land on bicycle or treadmill ergometers were analysed. If oxygen in the breathing gas was enriched by 30% or more exercise performance was increased (Mallette et al., 2018). However, in these studies the breathing gas supply was not in the focus. Because of this, any effects of hyperoxia on V̇_E_ remained unreported.

Thus, some single studies are presented to underline the ergogenic effects of hyperoxia by providing different parameters of performance and V̇_E_ in particular. One such parameter is time to exhaustion that is prolonged by hyperoxia (Adams and Welch, 1980) (Plet et al., 1992) (Ohya et al., 2016) (Mallette et al., 2018), Likewise, endurance was increased (Amann et al., 2008) together with the maximal power output (Ulrich et al., 2017) (Manselin et al., 2017). Other results would suggest an increased maximal aerobic power (Manselin et al., 2017), and a delayed onset of anaerobic metabolism, respectively (Miyamoto and Niizeki, 1995). Finally, while exercising and breathing EANx, V̇_E_ was decreased compared with matched loads and breathing air (Miyamoto and Niizeki, 1995) (Ulrich et al., 2017). Similar reductions in V̇_E_ were also reported from handgrip exercise during hyperoxia (Pokorsko et al., 1990). As a consequence of a reduced V̇_E_ at comparable work loads in hyperoxia and normoxia, dyspnoea perception was less during hyperoxia (Chronos et al., 1988) (Ulrich et al., 2017).

A quite different study from the professional world is added. Firefighters performed submaximal work employing self-contained breathing apparatuses. Breathing EAN40 compared with air, reduced V̇_E_ by 10% together with a reduced perceived breathing distress (Eves et al., 2002).

The beneficial hyperoxic effects on V̇_E_ and power output in healthy subjects might also apply to patients with pulmonary diseases. During exhausting leg work, asthmatics inhaled either air or 100% oxygen, the latter producing significantly less obstruction and a lower V̇_E_ (Resnick et al., 1979). Similarly, V̇_E_ in COPD patients was significantly less when breathing 100% oxygen than when breathing air during exercising on the same work load (Scano et al., 1982). In another group of COPD patients, hyperoxia clearly improved exercise endurance (O'Donell et al., 2001). In patients with interstitial lung disease (ILD), exercise-induced increases in V̇_E_ were attenuated by breathing 100% oxygen during exercise testing, demonstrating both a decreased ventilatory drive and an increased efficiency at achieved work loads (Cournoyer et al., 2020). These results suggest that hyperoxia could enhance the ability of patients with pulmonary diseases to train at higher work loads, resulting in more effective rehabilitation.

## 3 Increased pO_2_ and V̇_E_

Studies on the effects of increased pO_2_ values on V̇_E_ in the context with diving are scarce. In one own study, volunteers performed exercise during dry dives in a hyperbaric chamber. At comparable workloads, V̇_E_ at an ambient pressure of 3 bar (pO_2_ = 0.63 bar) was about 20% lower than at 1 bar (Kramkowski et al., 2021). In another study, divers performed exercise in the wet part of a hyperbaric chamber at 1 bar and 4.7 bar (pO_2_ = 1.0 bar). Hyperoxia under these circumstances reduced V̇_E_ by roughly 15% compared with normoxia (Peacher et al., 2010). It is mentioned that in contrast with these two studies, pO_2_ in EANx gases is not elevated due to the increased ambient pressure but due to the O_2_ enrichment.

Inspired by the latter two studies, two own studies were performed. (1) Recreational divers performed 40-min open-water dives to 25 m depth using air or EAN40. Apart from better preserved cognitive properties, V̇_E_ was significantly reduced by 12% with the hyperoxic breathing gas (Zenske et al., 2019). (2) To underscore the latter results, an earlier study with recreational divers under on-land conditions is cited. In an underwater ergometry system, scuba divers needed to fin-swim within a hexagonal parcours at a depth of 4 m with incrementally increased velocities until exhaustion (Steinberg et al., 2011). In concert with our previous study, V̇_E_ with EAN40 on the higher velocities was significantly reduced by 20% compared with air (Möller et al., 2021).

## 4 Mechanisms

Hyperoxic gas mixtures improve exercise tolerance in humans. In addition, V̇_E_ of these gases is reduced compared with normoxic gases. However, the underlying mechanisms remain unclear: thus, different hypotheses exist that, at least in part, are not convincing.

The O_2_ consumption during hyperoxic exercise (EAN30) was increased over that during normoxic exercise suggesting changes in skeletal muscle fibre type recruitment or in metabolic response (Prieur F. et al., 2002). This, however, would rather shorten the duration of oxygen availability. Based on similar results but employing EAN60, a shift toward increased fatty acid metabolism in the hyperbaric test was suggested (B. A. Wilson et al., 1975). However, a shift from glucose to fatty acid metabolism reduces the amount of energy produced per mole of oxygen consumed. Another hypothesis suggests that with a decreased V̇_E_ the cost of breathing is decreased resulting from the relative hypoventilation in hyperoxia (G. D. Wilson and Welch, 1980). Breathing work contributes approximately 2% to whole body oxygen consumption under resting conditions and may increase to 20% during very heavy workload (Thews, 1995). Data from our own studies nicely fit into this range: 12% reduction during dives on 25 m (Zenske et al., 2019), 20% reduction during heavy work at hyperbaric conditions (Kramkowski et al., 2021), and 20% during dives at high velocities (Möller et al., 2021). It is therefore not entirely clear, whether this effect can fully account for the observed extension of diving duration. According to a fourth hypothesis, hyperoxia enhances performance through a more efficient pulmonary gas exchange and a greater availability of oxygen to muscles and the brain (cerebral motor and sensory neurons) (Ulrich et al., 2017). Obviously, an improved O_2_ diffusion to the muscle will delay the metabolic acidosis from lactate accumulation (Miyamoto and Niizeki, 1995). However, enhanced steady state lactate concentrations are expected only at severe workload and therefore would not apply for calm recreational diving. Nevertheless, other studies have reported on decreased lactate accumulation during hyperoxic exercise (B. A. Wilson et al., 1975) (Stellingwerff et al., 2006). In turn, muscle efficiency appears to be already optimized in normoxia and is unlikely to contribute to the well-established improvement in exercise capacity induced by hyperoxia (Layec et al., 2015). Finally, it was described that cerebral oxygenation is limiting the exercise performance. Thus, a better cerebral oxygenation is suggested to be responsible for the improved performance with hyperoxia (Oussaidene et al., 2013). In addition, heart rate is decreased with hyperoxia thereby decreasing myocardial load (Wunderlich et al., 2017). After all, not only one single mechanism might be involved.

## 5 Conclusion

The roughly 75-year old contention must be rejected that hyperoxia reduces V̇_E_ could be explained by psychological factors (Bean, 1945). Numerous human studies with different degrees of hyperoxia, employing both cycle and treadmill ergometry, have shown reduced V̇_E_ during hyperoxic settings together with beneficial effects on power output, time to exhaustion and dyspnoea perception. Different hypotheses are provided that try to explain the underlying mechanism that might well be not a single one.

Acceptedly, hyperoxia is associated with an increased oxidative stress that might impair the CNS, the lungs and blood vessels of a diver. In case, instructions are followed, e.g. maximum pO_2_ ≤ 1.4 bar is followed, CNS oxygen toxicity will not become a major challenge. Then, advantages of EANx over air prevail like less narcotic effects, i.e. maintained cognitive performance, less bubble formation, and a better preserved sympathovagal balance.

Although several studies show that diving with EANx supply reduces minute ventilation, the mechanism(s) which may cause this effect are less clear. A potential mechanism may be a reduction of breathing work when supplying a gas with enhanced oxygen fraction. Although our data point in this direction, it is still unclear, whether this reduced breathing work may fully explain the reduction in minute volume. Such a reduction with O_2_-enriched gases has significant practical implications contributing to increase the safety of SCUBA diving, in particular in delaying unexpected running out-of-gas.

## Author contribution

We submit a short communication, where articles are included that were already published earlier. Therefore, we understand that the otherwise useful CRediT author statements do not apply here.

## Declaration of competing interest

The authors declare that they have no known competing financial interests or personal relationships that could have appeared to influence the work reported in this paper.
